# Involvement of the optic nerve in mutated *CSF1R*-induced hereditary diffuse leukoencephalopathy with axonal spheroids

**DOI:** 10.1186/s12883-016-0694-0

**Published:** 2016-09-13

**Authors:** Yaqing Shu, Ling Long, Siyuan Liao, Jiezheng Yang, Jianfang Li, Wei Qiu, Yu Yang, Jian Bao, Aiming Wu, Xueqiang Hu, Zhengqi Lu

**Affiliations:** 1 Department of Neurology, The Third Affiliated Hospital of Sun Yat-sen University, 600 Tianhe Road, Guangzhou, 510630 China; 2 Department of Ophthalmology, The Third Affiliated Hospital of Sun Yat-sen University, Guangzhou, 510630 China; 3 Department of Nuclear Medicine, The Third Affiliated Hospital of Sun Yat-sen University, Guangzhou, 510630 China

**Keywords:** Leukoencephalopathy, Colony-stimulating factor 1 receptor, Peripapillary retinal nerve fiber layer, Hereditary diffuse leukoencephalopathy with axonal spheroids

## Abstract

**Background:**

Hereditary diffuse leukoencephalopathy with axonal spheroids (HDLS) is a rare autosomal dominant disorder characterized by cerebral white matter degeneration and caused by mutations in the *colony-stimulating factor 1 receptor* (*CSF1R*) gene. Involvement of the optic nerves in hereditary diffuse leukoencephalopathy is rare.

**Case presentation:**

We report the case of a 30-year-old Chinese woman with HDLS, who carried a heterozygous c.2345 G > A (p.782Arg > His) mutation in exon 18 of *CSF1R*. She developed a gradual decline in motor ability, as well as cognitive and visual function, over the course of 4 months. Brain T2 fluid-attenuated inversion recovery-weighted magnetic resonance imaging revealed high signal lesions in the bilateral frontoparietal and periventricular deep white matter. Optical coherence tomography showed that the right peripapillary retinal nerve fiber layer was atrophic in the temporal quadrant while the left peripapillary retinal nerve fiber layer was thin in the temporal superior quadrant.

**Conclusions:**

A diagnosis of HDLS should be considered in patients with white matter lesions and optic nerves injury upon magnetic resonance imaging that mimics progressive multiple sclerosis.

## Background

Hereditary diffuse leukoencephalopathy with axonal spheroids (HDLS) is a rare autosomal dominant disorder characterized by cerebral white matter degeneration with axonal spheroids with variable clinical presentations, including personality and behavioral changes, dementia, depression, parkinsonism, and seizures. HDLS was first identified in a Western Swedish family reported in 1984 [1] and is caused by mutations in the *colony-stimulating factor 1 receptor* (*CSF1R*) gene [2]. HDLS appears as white matter lesions in a frontal predominant distribution upon brain magnetic resonance imaging (MRI), spreading out from the periventricular and deep white matter into the subcortical areas with enlarged ventricles and often signal changes in the corpus callosum [3, 4]. However, involvement of the optic nerves has not been previously reported in HDLS. We describe a HDLS patient who also had optic nerve lesions.

## Case presentation

**Fig. 1:**
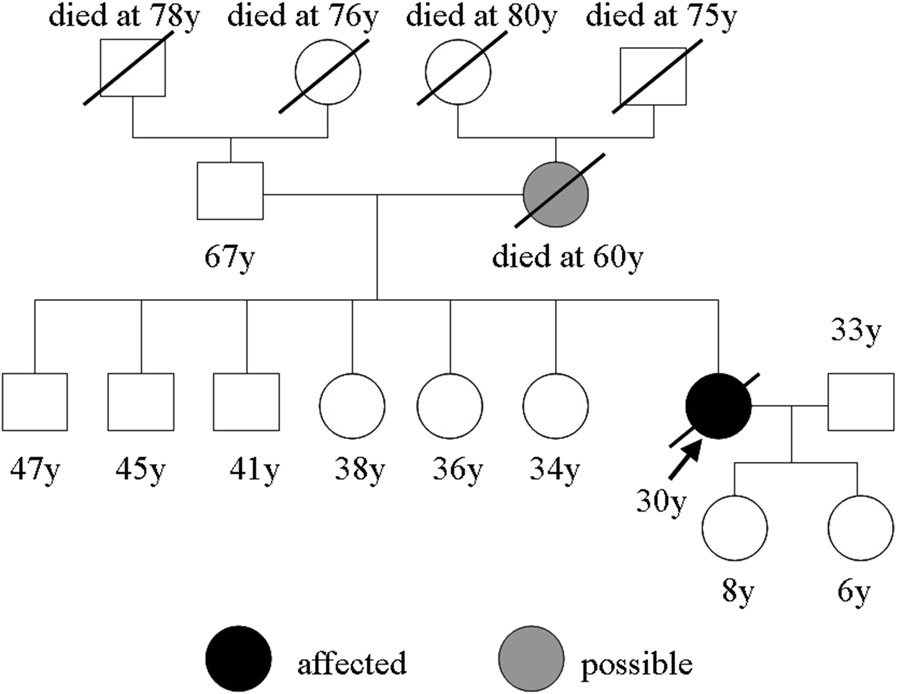
Family pedigree. The arrow indicates the proband (present patient). Her mother developed a motor disorder at 40 years of age and died at 60 years of age. Her grandparents, father, brothers, sisters, and daughters were not affected

A 30-year-old, non-smoking woman was referred to our hospital in August 2015, with a 4-month history of progressive lower limb weakness, gait disturbance, and visual disorder. Her mother experienced right lower limb weakness at 40 years of age, followed by progressive cognitive and motor deterioration and death at 60 years of age. The case family pedigree is shown in Fig. 1.

Prior to admission to our hospital, the patient exhibited neuropsychiatric symptoms, such as paraphasia and hallucination. She was diagnosed with multiple sclerosis (MS) and received immunotherapy with intravenous methylprednisolone at another hospital. However, her motor function progressively declined and she experienced frequent falls. When she was admitted to our hospital, her mini-mental state examination (MMSE) score was 25/30, with a Montreal cognitive assessment scale (MoCA) score of 17/30. The activities of daily living (ADL) score was 50/100 and the Morse fall risk factors assessment scale score was 45. Bilateral visual acuity was 0.8, muscle tone in her lower limbs increased, and muscle strength of the lower limbs was grade 4. She developed a spastic gait and an unsteady broad-based gait. Romberg’s sign, Babinski’s sign, and Chaddock’s sign were all positive.

Most laboratory tests were normal/negative, including the serum erythrocyte sedimentation rate, C-reactive protein, vitamins (B1\B2\B6\B9\B12), glycosylated hemoglobin, thyroid function, serum copper, ceruloplasmin, anti-nuclear antibody, paraneoplastic antibodies, and antibodies specific for the following: myelin oligodendrocyte glycoprotein, myelin-associated glycoprotein, myelin basic protein, and aquaporin-4. Serum treponema pallidum antibody, rapid plasma reagin, tolulized red unheated serum test, and human immunodeficiency virus antibody were all negative. Cerebrospinal fluid tests were normal, including negative oligoclonal IgG bands.

**Fig. 2:**
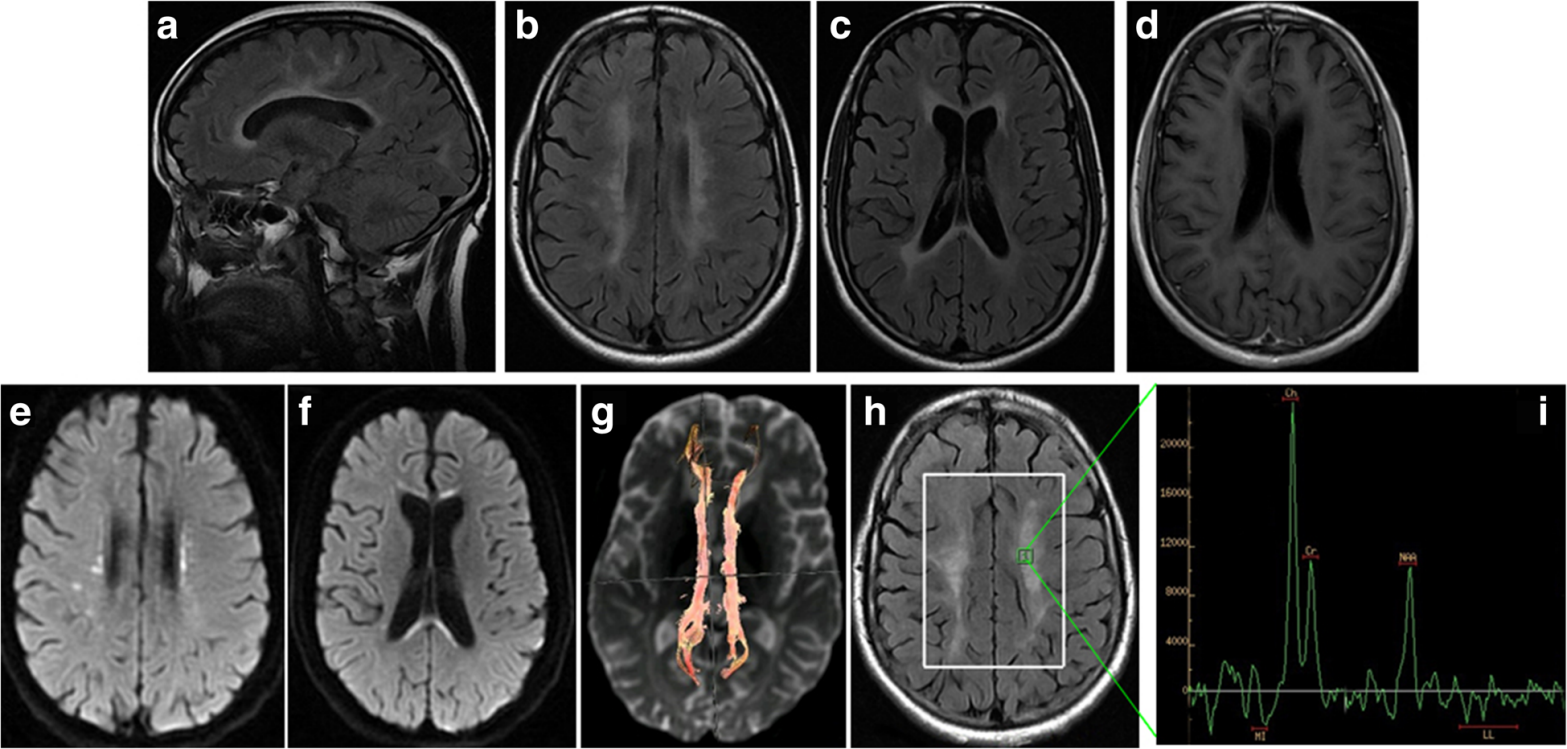
Brain MRI, DWI, DTI, and MRS images. T2/Flair showed multifocal periventricular white matter lesions (**a b** and **c**), without enhancement (**d**). DWI shows high-signal intensities in periventricular white matters and corpus callosum (**e f**). DTI shows decreased numbers of corpus callosum fibers, while subcortical arcuate fibers are spared (**g**). MRS shows increased Cho levels, while NAA levels are decreased in the white matter lesions (**h i**)

Brain T2 fluid-attenuated inversion recovery (FLAIR) MRI showed multiple, asymmetrical, hyperintense lesions, which appeared in periventricular areas and the white matter regions of the frontal and parietal lobes. Diffusion-weighted imaging (DWI) showed small dots of diffusion restriction within the white matter lesions. Magnetic resonance spectroscopy (MRS) showed increased levels of choline (Cho), although N-acetylaspartate (NAA) was decreased. Diffusion tensor imaging (DTI) revealed corpus callosum fiber loss, although fibers of the bilateral frontal, parietal, occipital, and temporal lobe and brainstem fibers were spared (Fig. 2).

**Fig. 3:**
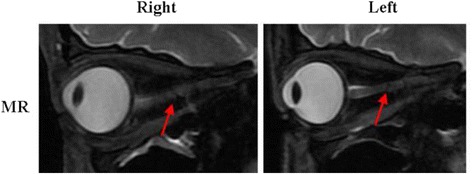
Optic nerves on MRI, showing that bilateral optic nerves are injured (red arrows)

**Fig. 4:**
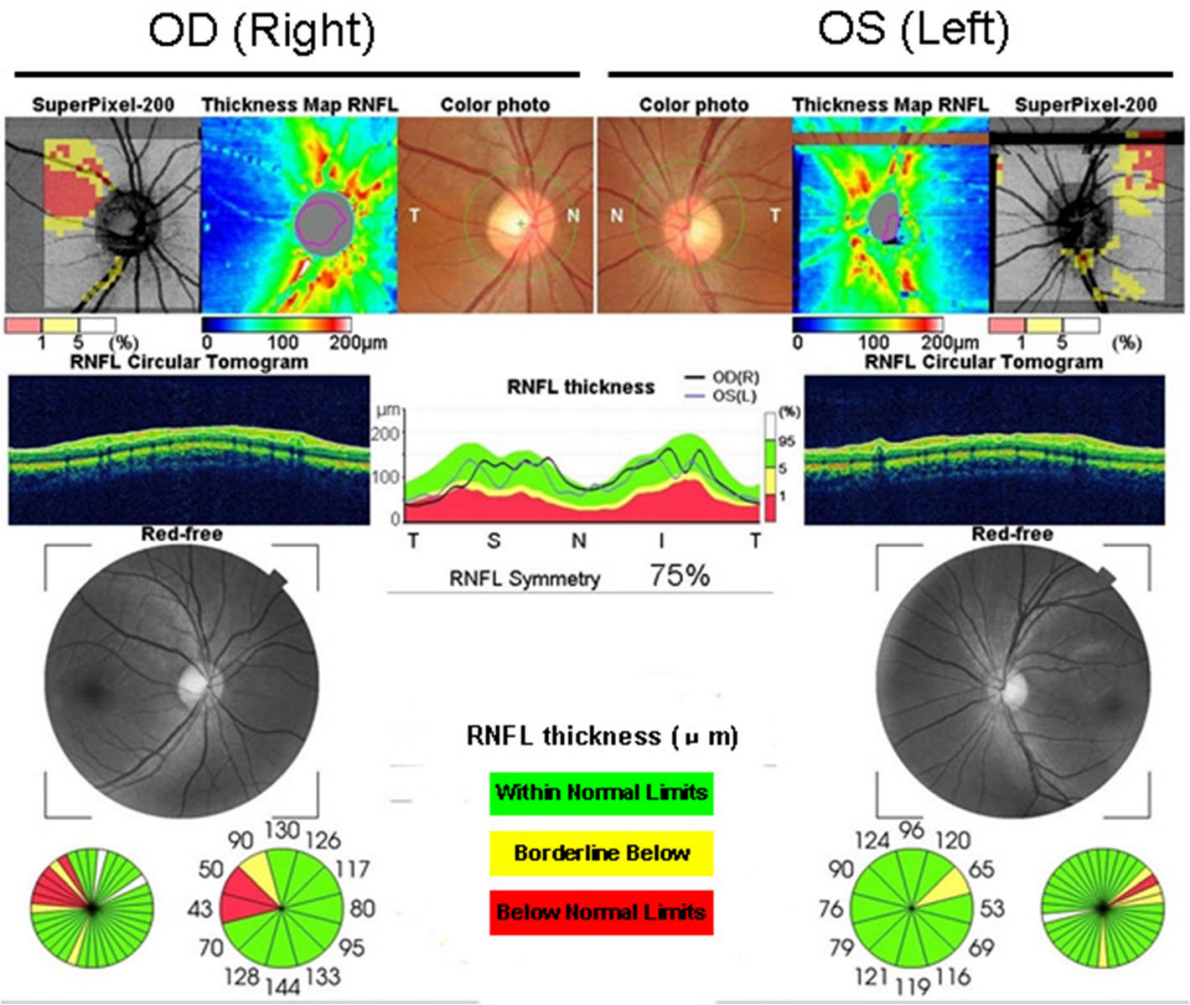
OCT shows that the right peripapillary retinal nerve fiber layer (pRNFL) is atrophic in the temporal quadrant, and the left pRNFL is thinning in the temporal superior quadrants. Green represents pRNFL thickness, which is within normal limits; yellow represents pRNFL thickness, which is below borderline; red represents pRNFL thickness, which is below normal limits

**Fig. 5:**
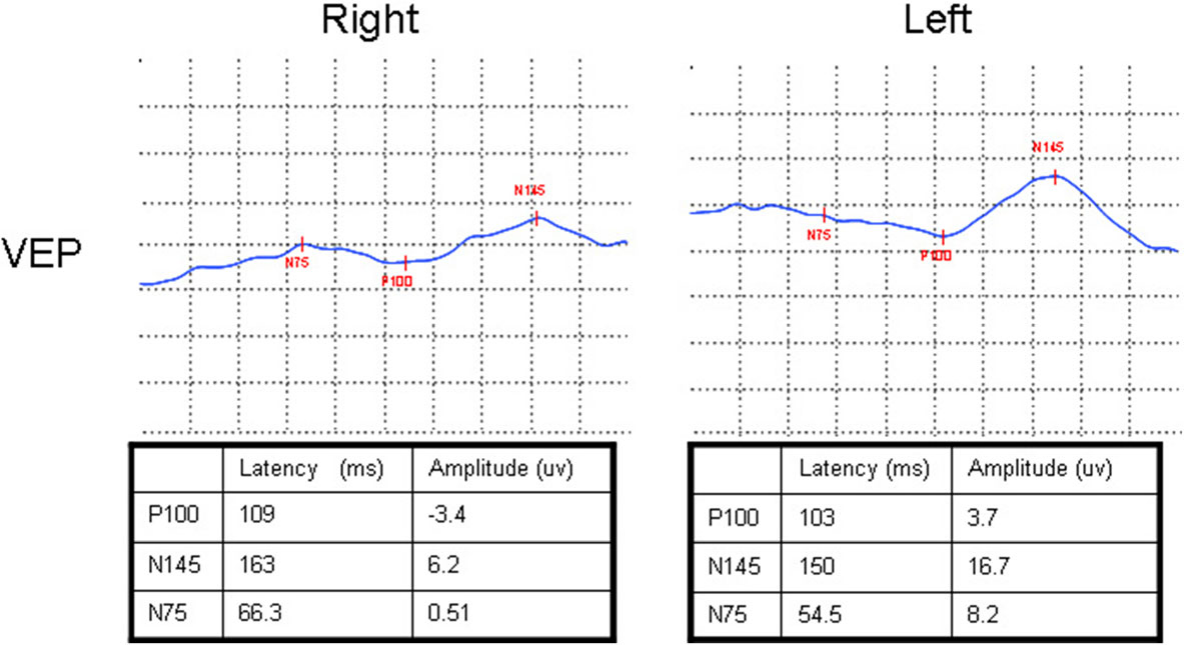
VEP shows reduced bilateral P100 amplitudes, although P100 latencies are normal in both eyes

**Fig. 6:**
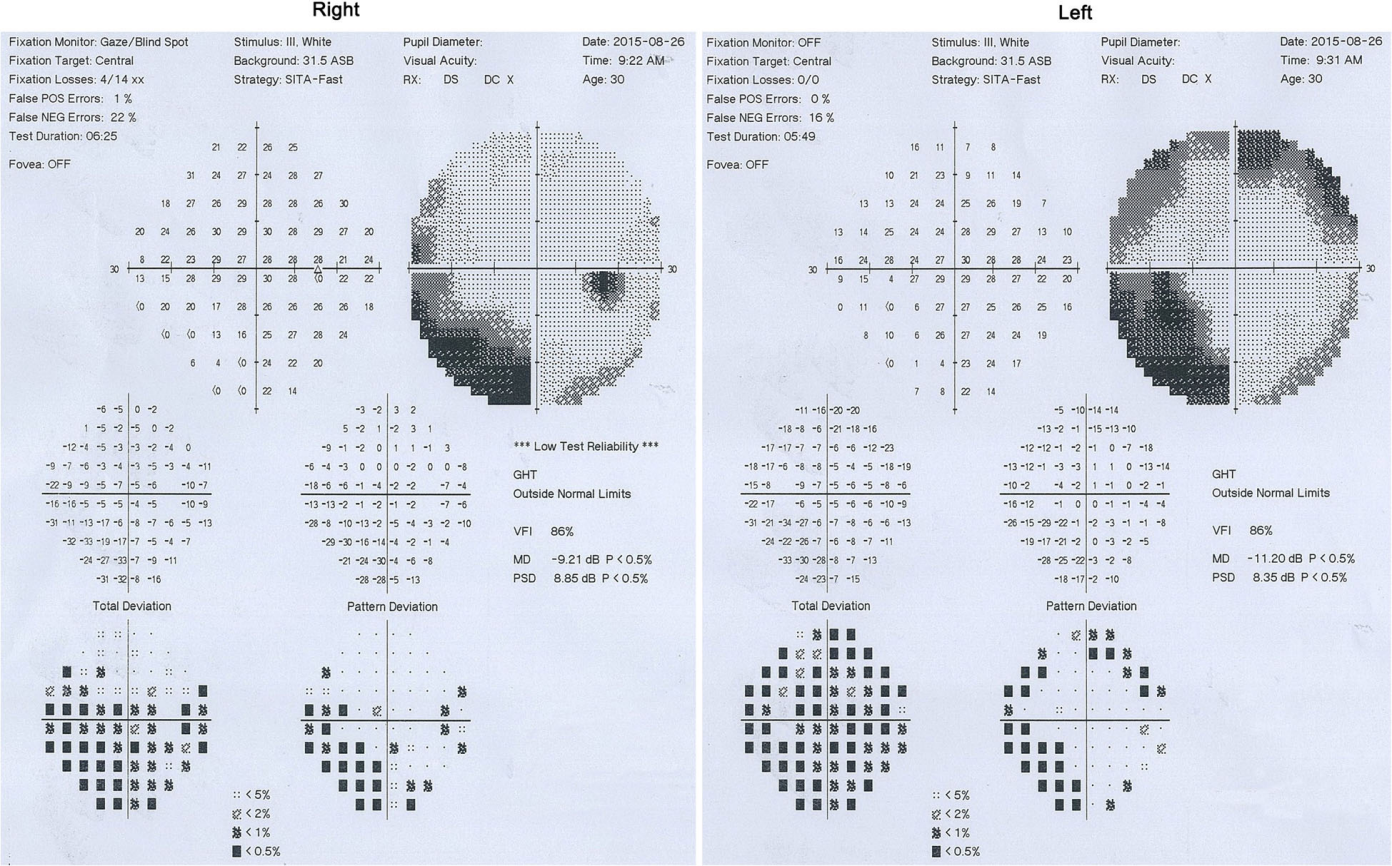
Visual fields in the right eye are partially missing in the upper right, lower right, and lower left quadrants, especially in the lower right quadrant. Visual fields in the left eye are partially missing in the four quadrants, especially in the upper left and lower right quadrants

Optic nerve MRI demonstrated bilateral optic nerves lesions (Fig. 3). Optical coherence tomography (OCT) showed that the right peripapillary retinal nerve fiber layer (pRNFL) was atrophic (pRNFL thickness 43–50 μm) in the temporal quadrant, and the left pRNFL was thin (65 μm) in the temporal superior quadrant (Fig. 4). Average RNFL thickness in healthy controls has been shown to be 93–108 μm [5]. Visual-evoked potential (VEP) showed reduced amplitudes of bilateral P100, although latencies of bilateral P100 were normal (Fig. 5). Bilateral intraocular pressures (both 15 mmHg) were normal. The visual fields of both eyes were partially missing in the different quadrants (Fig. 6).

**Fig. 7:**
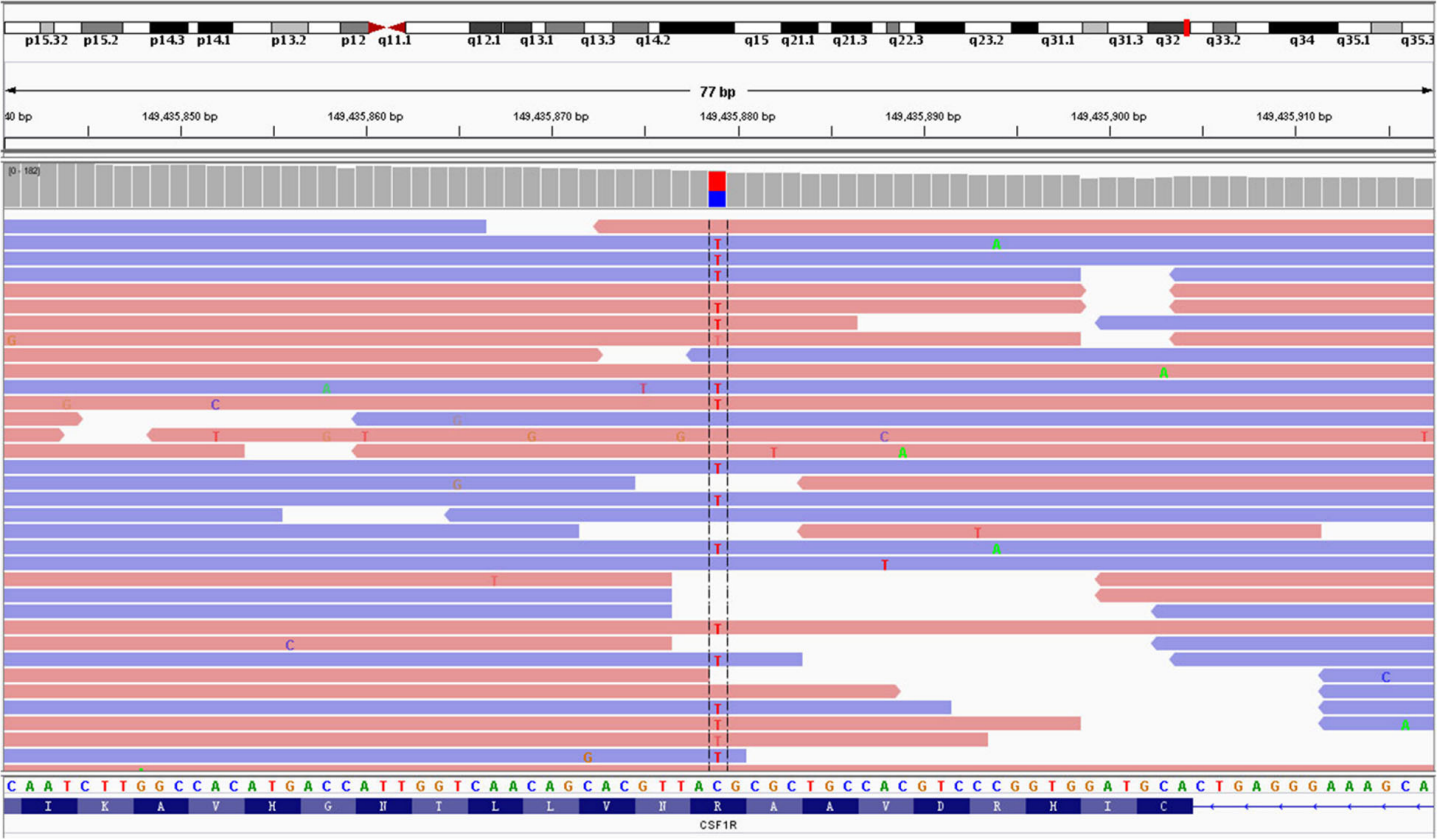
Gene analysis of *CSF1R*. The sequencing result from exon 18 of *CSF1R* (NM_005211.3) indicates a heterozygous c.2345 G > A (p.782Arg > His) substitution in the patient

Methylprednisolone therapy with two treatment courses (1000 mg/day for 5 days and 1000 mg/day for 3 days) resulted in no improvement. Genetic studies for leukodystrophy-related genes showed that a c.2345 G > A (p.782Arg > His) mutation in exon 18 of *CSF1R* was present (Fig. 7), which has been proven to be the pathogenic gene for HDLS. However, a brain biopsy was refused by the patient and her family. Seven months after discharge from our hospital, she showed progressive decline and reached a vegetative state and died on May 18, 2016.

The present case describes a patient with a genetically confirmed HDLS and a heterozygous c.2345 G > A (p.782Arg > His) mutation in exon 18 of the *CSFR1*, which was previously described by Kinoshita et al. [6]. HDLS has an autosomal dominant pattern of inheritance, and it may be sporadic. In our case, we presume that the patient’s mother was affected, because of early-onset motor impairment over the course of her illness. Unfortunately, however, there were no clinical data or brain biopsy samples from her mother.

In the present case, the patient experienced visual impairment and optic nerve impairment, which made it difficult to differentiate the disease from MS. Optic nerve injury lacking gadolinium enhancement was revealed by brain MRI, and the right temporal pRNFL was atrophic and left temporal superior pRNFL was thin upon OCT. To the best of our knowledge, involvement of the optic nerves has never been reported in HDLS, and the mechanism of optic nerve impairment in HDLS remains unknown. HDLS is pathologically characterized by non-inflammatory myelin loss, reactive astrocytosis, and axonal spheroids. Axonal spheroids of peripheral neuropathy have been found in HDLS, suggesting more widespread nervous system involvement [7]. We hypothesized that non-inflammatory myelin loss and axonal spheroids might also be involved in the optic nerves in our HDLS case. Additionally, OCT, which has emerged as a novel tool to depict neuro-axonal damage in MS by measuring non-myelinated central nervous system axons in the retina [5, 8], was also used to analyze RNFL thickness that is the better parameter for monitoring longitudinal axonal damage [9]. RNFL thickness has also been linked to metabolic signs of neurodegeneration in the visual cortex [10]. Several reports of MS patients have correlated RNFL atrophy with brain atrophy in patients with and without optic neuritis [8, 9, 11]. In our case, brain MRI showed multiple lesions in the white and gray matter, and DTI revealed lost or spared white and gray matter fibers. These results suggested that RNFL atrophy in OCT might be associated with brain abnormalities in HDLS. We hypothesized that the features revealed by OCT and neuroimaging might be related to axonal and myelin destruction in white matter lesions in HDLS.

The clinical features in the present case predominantly included progressive gait disturbance and cognitive and motor impairment that mimicked progressive MS, although the patient also exhibited atypical parkinsonian features, including bradykinesia and rigidity, which have been previously reported [12–14]. Although the mechanisms of atypical Parkinsonism remain unclear, it has been suggested that the disconnected fiber tracts in white matter lesions are structurally or functionally critical for performing movements [15, 16]. Subcortical and periventricular white matter lesions might interfere with ascending thalamocortical and descending corticospinal fibers, and a large number of white matter lesions have been observed in HDLS.

## Conclusions

Results from the present case study suggest that optic nerves can be involved in HDLS. RNFL atrophy observed by OCT might be associated with brain abnormalities in HDLS. Molecular analysis of the *CSF1R* gene should be performed when HDLS clinically and radiologically mimics progressive multiple sclerosis.

## Abbreviations

ADL: Activities of daily living
Choline: Cho
*CSF1R*: Colony stimulating factor 1 receptor
DTI: Diffusion tensor imaging
DWI: Diffusion weighted imaging
HDLS: Hereditary diffuse leukoencephalopathy with axonal spheroids
MMSE: Mini-mental state examination
MoCA: Montreal cognitive assessment scale
MRI: Magnetic resonance imaging
MRS: Magnetic resonance spectroscopy
MS: Multiple sclerosis
NAA: N-acetylaspartate
OCT: Optical coherence tomography
pRNFL: Peripapillary retinal nerve fiber layer
T2 FLAIR: T2 fluid-attenuated inversion recovery
